# The socioeconomic impacts of fibrodysplasia ossificans progressiva: evidence from a retrospective case-control study in France

**DOI:** 10.1093/jbmrpl/ziag125

**Published:** 2026-08-13

**Authors:** Geneviève Baujat, Stéphane Bouée, Oyinkan Solanke, Kim Croskery, Justin Kirion, Valérie Cormier-Daire, Anne-Sophie Jannot, Viviane Jeanbat, Thomas Funck-Brentano

**Affiliations:** Service de Médecine Génomique des Maladies Rares, Université Paris Cité, INSERM UMR 1163, Institut Imagine, Hôpital Necker-Enfants malades, Paris 75015, France; CEMKA, Boulevard Maréchal Joffre, Bourg-La-Reine 92340, France; Medical Affairs Rare Disease / Global HEOR, Medical Affairs Rare Disease / Global HEOR, Ipsen, London W2 1AF, United Kingdom; Medical Affairs Rare Disease / Global HEOR, Medical Affairs Rare Disease / Global HEOR, Ipsen, London W2 1AF, United Kingdom; CEMKA, Boulevard Maréchal Joffre, Bourg-La-Reine 92340, France; Service de Médecine Génomique des Maladies Rares, Université Paris Cité, INSERM UMR 1163, Institut Imagine, Hôpital Necker-Enfants malades, Paris 75015, France; Banque Nationale de Données Maladies Rares (Banque Nationale de Données Maladies Rares (BNDMR), Greater Paris University Hospitals (APHP), Paris 75012, France; CEMKA, Boulevard Maréchal Joffre, Bourg-La-Reine 92340, France; Department of Rheumatology, Lariboisière Hospital, APHP, Nord, Université Paris Cité, Paris 75010, France; BIOSCAR, Université Paris Cité, Inserm, Paris 75010, France

**Keywords:** fibrodysplasia ossificans progressiva, French National Healthcare System Database, French National Rare Diseases Registry, prevalence, healthcare resource utilization, socioeconomic costs, comorbidities, mortality

## Abstract

Fibrodysplasia ossificans progressiva (FOP) is an ultra-rare genetic bone disorder associated with considerable clinical, economic, and societal impacts on patients, their caregivers, and the healthcare system. The aim of this study was to update the estimated prevalence of FOP in France, characterize the FOP patient population and impact of the disease, assess associated mortality rates, and quantify healthcare resource utilization and socioeconomic costs for individuals with FOP. An observational, retrospective case-control study was conducted using linked data for individuals with FOP from the French National Rare Diseases Registry and the French National Healthcare System Database (SNDS). Results were compared with the SNDS for a control group of individuals with any disease other than FOP. Prevalence of FOP was estimated at 1.39 per million. Data were available for 77 individuals with FOP and 769 controls without FOP. Of 70 individuals with FOP and a social security number at the index date, 50.0% (*n* = 35/70) were female, and the mean (SD) age was 25.3 (15.7) yr. Comorbidities were consistently more prevalent in individuals with FOP than in people without FOP. Consultations with general practitioners and non-physician healthcare providers, hospitalizations, and use of medical devices and treatments were significantly greater in individuals with vs without FOP (*p* < .05 for all). Mean total annual healthcare expenditure was more than 10 times greater in individuals with FOP than those without FOP from a societal perspective (largely driven by outpatient costs) and 14 times greater from a payer perspective. Overall survival was significantly worse in individuals with FOP than in individuals without (*p* < .0001). Findings from this study reinforce the clinical, economic, and societal impacts associated with FOP. Improving disease awareness and developing new interventions to support a reduction in these impacts should be considered key priorities for the FOP community and healthcare providers.

## Introduction

Fibrodysplasia ossificans progressiva (FOP) is an ultra-rare genetic disorder, characterized by congenital skeletal malformations of the hallux (great toe), with cumulative and irreversible episodic formation of extraskeletal bone tissue (heterotopic ossification; HO) in soft and connective tissues.^1^ In FOP, HO typically affects the upper body and cranial regions before the lower body and more distal regions.^2^^,^^3^ HO progressively restricts movement, leading to cumulative disability, deformities, reduced quality of life (QoL), and shortened life expectancy.^4–8^ Other symptoms include inflammatory flare-up episodes that often lead to reduced movement and stiffness and promote HO formation. Pain may occur before, during, and independently of flare-ups, demonstrating that pain is not limited to periods of active flare activity.^9^ These flare-ups can occur in response to trauma, injections, or viral infections but can also happen spontaneously.^6^ Flare-ups begin in early childhood, with most (93%) individuals with FOP experiencing symptoms before 18 yr of age,^10^ although physical impairments can manifest before the first decade of life.^11^ Flare-ups manifest more frequently in the upper body (neck, trunk, and upper limbs) before the age of 8 yr and in the lower body after age 8.^12^

Although a link with paternal age has been suggested,^13^^,^^14^ FOP occurs mostly as de novo cases; only a few examples of familial cases, with genetic transmission in an autosomal dominant manner, are known.^15^^,^^16^ FOP is caused by a pathogenic variant in the Activin A receptor, type I/Activin-like kinase 2 (*ACVR1/ALK2*), most commonly through a recurrent single-nucleotide substitution (c.617G > A; p.R206H) in the *ACVR1* gene.^17–19^ ALK2 is a BMP type I receptor that regulates skeletogenesis and skeletal growth.^18^ The pathogenic variants in *ACVR1* identified in patients with FOP to date are gain-of-function variants that cause hyperresponsiveness to the canonical ligand BMP, a “leaky tap” of nonliganded signaling, and de novo responsiveness to Activin A. While all are associated with FOP, different gene variants may result in distinct clinical presentations with differing effects on tissues and organs (including brain malformations/impairments [anatomic abnormalities of the cerebellum, cerebral cavernous malformation, cognitive impairment, craniopharyngioma, diffuse cerebral dysfunction with seizures]; reduction of multiple digits, thumb malformations, and absent fingernails/toenails; severe growth retardation; sparse scalp hair; persistence of primary teeth in adulthood; cataracts, retinal detachment, childhood glaucoma; polyostotic fibrous dysplasia; primary amenorrhea; aplastic anemia; and hypospadias).^20^ Animal models of HO that closely mimic the characteristic features of FOP, such as mouse models with a knock-in insertion of the R206H pathogenic variant, have facilitated histological and molecular study of the HO process. Results of these analyses showed that the process of spontaneous HO or HO occurring in response to injury involved (1) local inflammatory cell infiltration, (2) apoptosis of skeletal muscle cells, and (3) a robust fibroproliferative response, followed by (4) an endochondral ossification process and the formation of extraskeletal tissue.^18^

FOP is associated with considerable clinical, economic, and societal impacts, affecting individuals with FOP, their caregivers, and the healthcare system. This condition is characterized by significant interindividual variability. While most (56%-67%) individuals with FOP experience moderate-to-severe pain during flare-ups, approximately one-third of individuals with FOP report no-to-mild pain during flare-ups.^9^ In addition, a substantial proportion (30%-55%) of individuals with FOP also experience pain in the absence of HO and flare-ups.^9^ The pain associated with FOP can have adverse effects on mental health, with individuals with FOP reporting feelings of anxiety, depression, or irritability.^9^ Additionally, misdiagnosis and underdiagnosis can lead to unnecessary and invasive procedures, such as biopsies, as well as increased psychological stress, contributing to the overall impacts of FOP.^21–23^

Individuals with FOP experience high rates of healthcare consultations, multidisciplinary care, and hospitalization. The most common reasons for hospital admissions are pain management, fall/injury/accident/fracture, and respiratory infection.^8^^,^^24^ In addition, the formation of extraskeletal bone tissue leads to progressive impacts on mobility, the ability to perform activities of daily living (ADLs), and basic self-care tasks such as bathing.^6^ Thus, individuals with FOP require additional resources for mobility, such as wheelchairs, and most will eventually require caregivers to assist with performing ADLs.^5^^,^^6^^,^^25^ The need for home modifications, caregivers to assist individuals with FOP with ADLs, and specialized transportation can add substantial economic impacts to individuals and their families.^25^ Disease progression may influence career choices and the ability to maintain regular employment, particularly among those with severe mobility restrictions. Family members and primary caregivers may also modify their employment or miss work to provide necessary support.^25^ Consequently, FOP creates considerable socioeconomic challenges for patients and caregivers, with caregiving linked to poorer health, reduced QoL, and productivity losses.^25^ The social impacts of FOP are also considerable. Children with FOP may miss school and be unable to participate in physical and social activities or sports because of joint limitations, deformities, and/or concerns that falls or trauma can lead to flare-ups and disease progression.^6^^,^^25^ Moreover, parents of children with FOP may need to provide various mobility aids (eg, electric wheelchair) and/or obtain an adapted car for travel.^6^ Additionally, family members may experience anxiety about their relative potentially experiencing flare-ups and disease progression and report the challenges of assisting a relative with FOP are very or extremely stressful.^25^

In 2012, the prevalence of FOP in France was estimated to be 1.36 per million residents.^2^ A study conducted in the US in 2020 determined the prevalence of FOP to be approximately 0.88 per million US residents, although this was considered likely to be an underestimate.^26^ Based on these differing reported prevalence rates, the International FOP Association (IFOPA) estimates FOP affects approximately 1 per 1 million persons.^27^ However, it is recognized that FOP can remain undiagnosed or be misdiagnosed, which could mean epidemiological data are incomplete.^21^^,^^28^ Consequently, there is a need to better understand the impact of disease in individuals with FOP, including its prevalence and the associated clinical and socioeconomic challenges. This understanding will help inform decision-making for various stakeholders, including individuals with FOP, clinicians, and payers. To address the unmet need of understanding the impact of FOP, the aim of this study was to generate an updated estimate of the prevalence of FOP in France, describe the characteristics of the patient population, examine mortality associated with FOP, and quantify healthcare resource utilization (HCRU) and socioeconomic costs for people living with FOP.

## Materials and methods

### Study design and data sources

**Figure 1:**
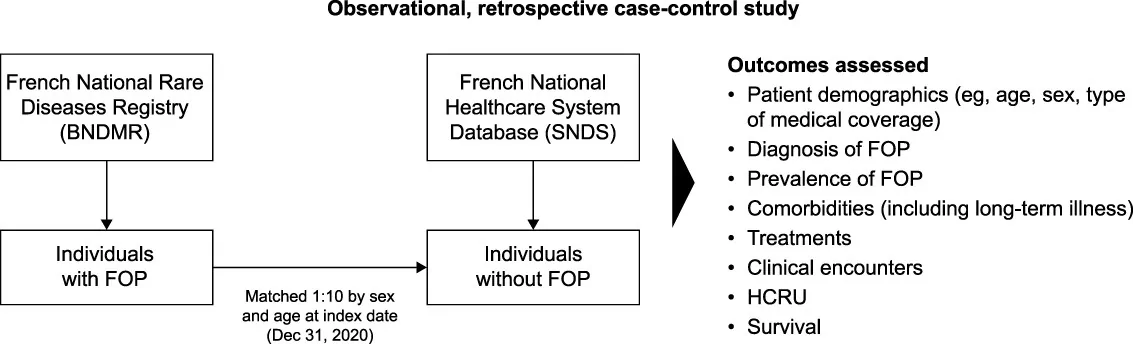
Study design. Abbreviations: FOP, fibrodysplasia ossificans progressiva; HCRU, healthcare resource utilization.

This observational, retrospective case-control study was conducted using deterministic matching with linked data on patients with and without FOP from the *Banque Nationale de Données Maladies Rares* (BNDMR; the French National Rare Diseases Registry), which included data on prevalence and disease characteristics of patients presenting rare conditions and followed by dedicated centers of expertise (*Centre de Reference Maladies Rares*), and the *Système National des Données de Santé* (SNDS; the French National Healthcare System Database), which provided HCRU data. Data of patients with FOP from the BNDMR were compared with a control group comprising individuals with any disease other than FOP from the SNDS (Figure 1). The BNDMR collects a common data set on all patients followed up in rare diseases expert centers and was progressively deployed beginning in 2008 and completely deployed in 2021.^29^ The aim of the registry is to improve the understanding of the burden of disease for rare conditions as well as the resources needed to treat those conditions.^29^ The common minimal data set that is collected for each patient encompasses administrative details, including date and place of birth, place of residency, patient demographics (eg, sex), and medical data such as diagnosis, familial or non-inherited de novo (ie, no family history) occurrence of FOP, age at first symptoms, age at diagnosis confirmation, and diagnostic confirmation method (clinical, molecular, or other), and date of death.

The SNDS includes exhaustive HCRU data for virtually all individuals covered by the French national health insurance system (over 66 million people living in France), equating to approximately 99% of the French population, establishing the SNDS as one of the largest claims databases in the world.^30^^,^^31^ The SNDS aggregates numerous comprehensive national health databases (claims database [Datamart de Consommation Inter Régimes; DCIR], hospital discharge database [Programme de Médicalisation des Systèmes d'Information; PMSI], and the causes-of-death register) and provides comprehensive data on pharmacoeconomics and pharmacoepidemiology.^30^ The SNDS captures anonymized, individual-level data on sociodemographic characteristics, comorbidities, hospitalizations, treatment, HCRU costs, medical device use, and mortality.^30^

Linking the BNDMR and SNDS facilitates a detailed analysis of the care pathways and HCRU attributable to FOP in France.^32^

### Study population and data management

Two distinct populations were included in the analyses:

1. Individuals with a diagnosis of FOP: these individuals were identified in the BNDMR based on Orphanet code 337, regardless of age or whether they were alive or deceased at the end of the observation period, and included those who had not declined to have their data from the SNDS analyzed. Each case of FOP was reviewed by the same clinician, who analyzed the associated clinical description, atypical features, and associated diagnoses. Individuals who had been misdiagnosed with FOP (eg, individuals with multiple osteochondromas or non-traumatic and traumatic myositis ossificans) were excluded from the analysis. Patients who were not living in France or were diagnosed after December 31, 2020, were also excluded. Individuals alive on January 1, 2021, were considered for the prevalence calculation and the description of the population, while all individuals with FOP were considered for the survival analysis. The cutoff date for study outcomes was December 31, 2020. Molecular confirmation was not mandatory for inclusion in the study.
2. The control group consisted of individuals not suffering from FOP randomly selected from the SNDS; data were matched to the FOP group exactly by year of birth and sex.

The previously estimated number of individuals with FOP in France was 89.^2^ Therefore, for each patient with FOP assessed, 10 individuals without FOP were included to increase the power of statistical comparisons.

The quality of the SNDS was ensured, controlled, and audited internally by the National Health Insurance Fund, and the reliability, precision, and safety of the PMSI were checked in each hospital by a physician responsible for medical information. At receipt of the data, the extraction criteria for individuals with FOP and controls, as well as the number of controls per patient, were checked. At the level of the study database, quality controls were applied on SNDS data for the detection and individualization of twins, exclusion of individuals without any healthcare consumption for more than 1 yr (beginning with the last available year), and deletion of any healthcare consumption after the date of death.

### Study outcomes

Study outcomes assessed were patient demographics, diagnosis of FOP, prevalence of FOP, comorbidities (including long-term illness, which was defined as severe or chronic in nature and requiring prolonged treatment, with individuals receiving 100% reimbursement on the basis of social security tariff), treatments received, clinical encounters, HCRU (hospitalizations, medical device use, and costs), and survival.

### Statistical analyses

Statistical analyses were performed using SAS software (SAS Institute, North Carolina), version 9.4.

Quantitative variables from the BNDMR and SNDS were analyzed using count, number of missing values, mean, SD, median, minimum, and maximum. Qualitative variables were described using the number of missing values, and the percentage of each modality was calculated on the responses expressed.

Comparative analyses were conducted on data collected from the SNDS for the FOP population and control group. For categorical variables, the chi-square test was applied; if theoretical sample sizes were <5, Yates continuity correction or Fisher exact test was used. For continuous variables, when distribution was close to normal (non-significant Shapiro-Wilk test), a Student’s *t* test or ANOVA was performed. If this was not the case, a Kruskal-Wallis non-parametric test was used.

Survival analyses were performed using the Kaplan-Meier method and log-rank test. Survival time was calculated (in years) from the date of first activity in the BNDMR (which was the date of first consultation at a rare disease center collecting data in the BNDMR) to the date of death or until December 31, 2022, for censored events.

FOP prevalence was estimated by dividing the number of individuals identified with FOP from the BNDMR alive on December 31, 2020 (index date, which was the date at which the most up-to-date data were available at the time of the study design), by the total population of France on that date (67 697 091).^33^ The 95% CI for prevalence was calculated using Wilson’s method with a continuity correction of a Poisson law.^34^ Comparative analyses between the FOP and control cohorts were based on data collected from the SNDS (HCRU, costs, comorbidities, and medical device use). To provide insights on the impacts of disease, subgroup analyses according to age and disease severity were also conducted. The Charlson Comorbidity Index (CCI) score was calculated according to the methodology from Bannay et al. to account for the specific characteristics of French data derived from the SNDS.^35^ The 17 comorbidities were identified in the year preceding the index date based on diagnoses and *Groupes Homogènes de Malades* (Homogeneous Groups of Patients) from *Médecine, Chirurgie, Obstétrique* (Medicine, Surgery, Obstetrics) hospitalizations (*International Classification of Diseases, Tenth Revision* [*ICD-10*] codes), long-term disease status, and reimbursements for treatments and medical procedures.

HCRU and costs were reported for 2019, except in special cases such as comorbidities or use of medical devices, which had to be detected for more than 1 yr to be relevant. Economic analyses were reported from societal and third-party/healthcare payer perspectives. A comparison of costs vs the control group provided a relative value of the increased costs associated with FOP management and care. Direct costs included ambulatory outpatient care (eg, physician services, dental care, paramedical care, laboratory and imaging services, pharmacy and medical devices, optical care, transportation, other outpatient care) available in the SNDS data and hospitalizations (private or public). The reimbursed amounts for each item used were aggregated to calculate the total annual cost per patient. The proportion of individuals receiving financial aid for a disability leading to a work impairment was directly available in the SNDS and included in the comparative analyses. The mean, SD, median, minimum, and maximum, and IQR of the first and third quartiles for each item were also calculated.

## Results

### Patient demographics

Based on the BNDMR, 94 individuals with FOP were alive on December 31, 2020. Thus, the prevalence was estimated at 1.39 per million (95% CI: 1.13-1.71). A total of 70 eligible individuals with FOP had a social security number available for linking to the SNDS. These 70 individuals were matched for age and sex to 699 individuals without FOP (control cohort; only 9 control individuals were identified for 1 patient with FOP) for the analyses. An additional 7 individuals with FOP without a social security number available were recorded in the BNDMR and followed until December 31, 2022. The absence of a social security number was related to incomplete data entry in the BNDMR by some clinicians. Patients without a recorded social security number did not differ from those with an available identifier. These 7 individuals were matched for age and sex to 70 controls without FOP. The total pool of 77 individuals with FOP and 769 controls without FOP was used to perform survival analyses.

Of the 70 individuals with FOP and a social security number at the index date, 50.0% (*n* = 35/70) were female, and the mean (SD) age was 25.3 (15.7) yr. As the controls were age- and sex-matched, there was no significant difference in the age and sex distribution in the control group (*n* = 699; 49.9% were female, and the mean [SD] age was 25.3 [15.6] yr). Based on age group at index date, most individuals with FOP (78.6%; *n* = 55) or without FOP (78.8%; *n* = 551) were aged ≥10 yr (Table 1).

**Table 1:** Patient demographics in the FOP (*n* = 70) and control (*n* = 699)^a^ cohorts.

	Individuals with FOP (*n* = 70)	Controls (*n* = 699) ^a^	*p*-value
**Sex, *n* (%)**			.9909
**Male**	35 (50.0)	350 (50.1)	
**Female**	35 (50.0)	349 (49.9)	
**Age at index date** ^**b**^			.9725
**Mean (SD), years**	25.3 (15.7)	25.3 (15.6)	
**Median (minimum/maximum, IQR), years**	22.5 (1.0/60.0, 13.0-41.0)	23.0 (1.0/60.0, 13.0-41.0)	
**Age group at index date,** ^**b**^ ***n* (%)**			.9999
**<10 yr**	15 (21.4)	148 (21.2)	
**10-20 yr**	14 (20.0)	139 (19.9)	
**20-30 yr**	15 (21.4)	152 (21.7)	
**≥30 yr**	26 (37.1)	260 (37.2)	
**Supplementary health insurance for solidarity for adults in 2019, *n* (%)**	7 (10.3)^c^	73 (13.0)^c^	.5284

Abbreviation: FOP, fibrodysplasia ossificans progressiva.

^a^For 1 individual with FOP, only 9 controls were identified. Controls did not have FOP.

^b^Index date: December 31, 2020.

^c^Percentages were calculated only for those individuals for whom data were available.

### Diagnosis of FOP

Most individuals with FOP had non-inherited, de novo cases (ie, no family history; 87.1%; *n* = 54; Table 2). In most patients, the first signs of FOP were identified later in life rather than at birth (79.7%; *n* = 51; Table 2). The mean (SD) age at first signs was 3.71 (4.86) yr, although 32 (50.0%) of the 64 individuals for whom data were available were aged <2 yr when the first signs of FOP were identified (Table 2). No data about the age of symptom onset were available for 6 individuals.

**Table 2:** First signs and diagnosis of FOP.

Characteristic	Individuals with FOP (*n* = 70)
**Inheritance, *n* (%)**	
**Unknown**	8
**Hereditary**	8 (12.9)^a^
**Non-inherited de novo**	54 (87.1)^a^
**Year of diagnosis, *n* (%)**	
**ND**	12
**<2008**	37 (63.8)^a^
**≥2008**	21 (36.2)^a^
**First signs, *n* (%)**	
**Unknown**	6
**At birth**	13 (20.3)^a^
**After birth**	51 (79.7)^a^
**Age at first signs, years**	
***n* (%)**	64 (91.4)
**Mean (SD)**	3.7 (4.9)
**Median (minimum/maximum, IQR)**	1.9 (0.0/23.0, 0.2-5.5)
**Age at first signs (class), *n* (%)**	
**ND**	6
**0-2 yr**	32 (50.0)^a^
**2-8 yr**	20 (31.3)^a^
**≥8 yr**	12 (18.7)^a^
**Diagnosis, *n* (%)**	
**Unknown**	10
**At birth**	3 (5.0)
**After birth**	57 (95.0)
**Age at diagnosis, years**	
***n* (%)**	58 (82.9)
**Mean (SD)**	6.4 (6.6)
**Median (minimum/maximum, IQR)**	4.0 (0.0/36.0, 1.5-9.0)
**Age at diagnosis (class), *n* (%)**	
**ND**	12
**0-2 yr**	16 (27.6)^a^
**2-8 yr**	23 (39.6)^a^
**≥8 yr**	19 (32.8)^a^
**Time between first signs and diagnosis, months**	
**Mean (SD)**	29.1 (52.1)
**Median (minimum/maximum, IQR)**	2.5 (−24.04^b^/264.0, 0.0-43.0)

Abbreviations: FOP, fibrodysplasia ossificans progressiva; ND, no data.

^a^Percentages were calculated only for those individuals with FOP for whom data were available.

^b^Diagnosis was sometimes made in the presence of minor signs (such as hallux valgus), but obvious clinical signs occurred later, resulting in a negative delay in 1 case.

Based on the age group at index date, the mean (SD) age at diagnosis was 2.43 (2.34) yr for individuals aged <10 yr at index date, 4.64 (4.59) years for individuals aged 10-20 yr, 5.91 (4.90) years for individuals aged 20-30 yr, and 9.29 (8.29) yr for individuals aged ≥30 yr (*p* = .0189 for the difference between age at diagnosis for age groups). The mean (SD) delay between first signs and diagnosis was numerically shorter in individuals aged <10 yr at the index date (8.6 [23.2] months) compared with the older age groups (30.4 [59.8]-39.3 [63.1] months), although this difference was not statistically significant (*p* = .5291; Table S1). Until the last 10 yr, FOP diagnosis was suspected based on clinical and radiological features and confirmed by FOP expert physicians within the French Center of Reference for Rare Bone Diseases. Consequently, the number of patients with molecular confirmation of FOP was not available in the BNDMR.

### Comorbidities

Comorbidities were consistently significantly more prevalent in individuals with vs without FOP, with mean (SD) CCI scores in 2019 of 0.3 (0.7) and 0.1 (0.3; *p* < .0001), respectively (Table 3). Almost one-quarter (22.9%; *n* = 16) of individuals with FOP had a CCI score ≥ 1 compared with 3% (*n* = 21) of controls (Table 3). Notable long-term comorbidities occurred significantly more frequently among individuals with FOP compared with the control group and included neurological and muscular disorders (21.4% vs 0.9%; *p* < .0001), malignant tumors (4.3% vs 0.9%; *p* = .0407), inherited metabolic diseases (4.3% vs 0%; *p* = .0007), systemic disease (2.9% vs 0.1%; *p* = .0231), and ankylosing spondylitis (2.9% vs 0%; *p* = .0082; Table 3).

**Table 3:** Comorbidities and long-term illness-related comorbidities in the FOP (*n* = 70) and control (*n* = 699) cohorts.

	Individuals with FOP (*n* = 70)	Controls (*n* = 699) ^a^	*p*-value
**CCI score in 2019, mean (SD)**	0.3 (0.7)	0.1 (0.3)	<.0001
**CCI score in 2019, *n* (%)**			
**0**	54 (77.1)	678 (97.0)	
**1 to 6**	16 (22.9)	21 (3.0)	
**Other comorbidities, *n* (%)** ^**b**^			
**Nutritional supplements**	29 (41.4)	13 (1.9)	<.0001
**Depression**	14 (20.0)	74 (10.6)	.0183
**Malnutrition**	7 (10.0)	1 (0.1)	<.0001
**Fractures leading to a hospital stay**	6 (8.6)	19 (2.7)	.0202
**Terminatory respiratory failure**	5 (7.1)	– (–)	<.0001
**Urinary lithiasis**	4 (5.7)	4 (0.6)	.0033
**Dental infections**	3 (4.3)	4 (0.6)	.0194
**Long-term disease groups, *n* (%)** ^**b**^			
**ALD code 0**^**c**^	34 (48.6)	9 (1.3)	<.0001
**Neurological and muscular disorders**	15 (21.4)	6 (0.9)	<.0001
**Malignant tumor**	3 (4.3)	6 (0.9)	.0407
**Inherited metabolic diseases**	3 (4.3)	– (–)	.0007
**Systemic disease (lupus, scleroderma, etc)**	2 (2.9)	1 (0.1)	.0231
**Ankylosing spondylitis**	2 (2.9)	– (–)	.0082

Abbreviations: ALD, *affection de longue durée* (long-term illness); FOP, fibrodysplasia ossificans progressiva; CCI, Charlson Comorbidity Index.

^a^Controls did not have FOP.

^b^Other comorbidities and long-term disease groups include only those occurring in statistically significantly higher percentages of individuals with FOP. Listed in descending order by occurrence in individuals with FOP.

^c^ALD code 0 means that individuals are covered for a condition that does not belong to the 30 ALDs recognized by the French Social Security system.

### Treatments and clinical encounters

**Figure 2:**
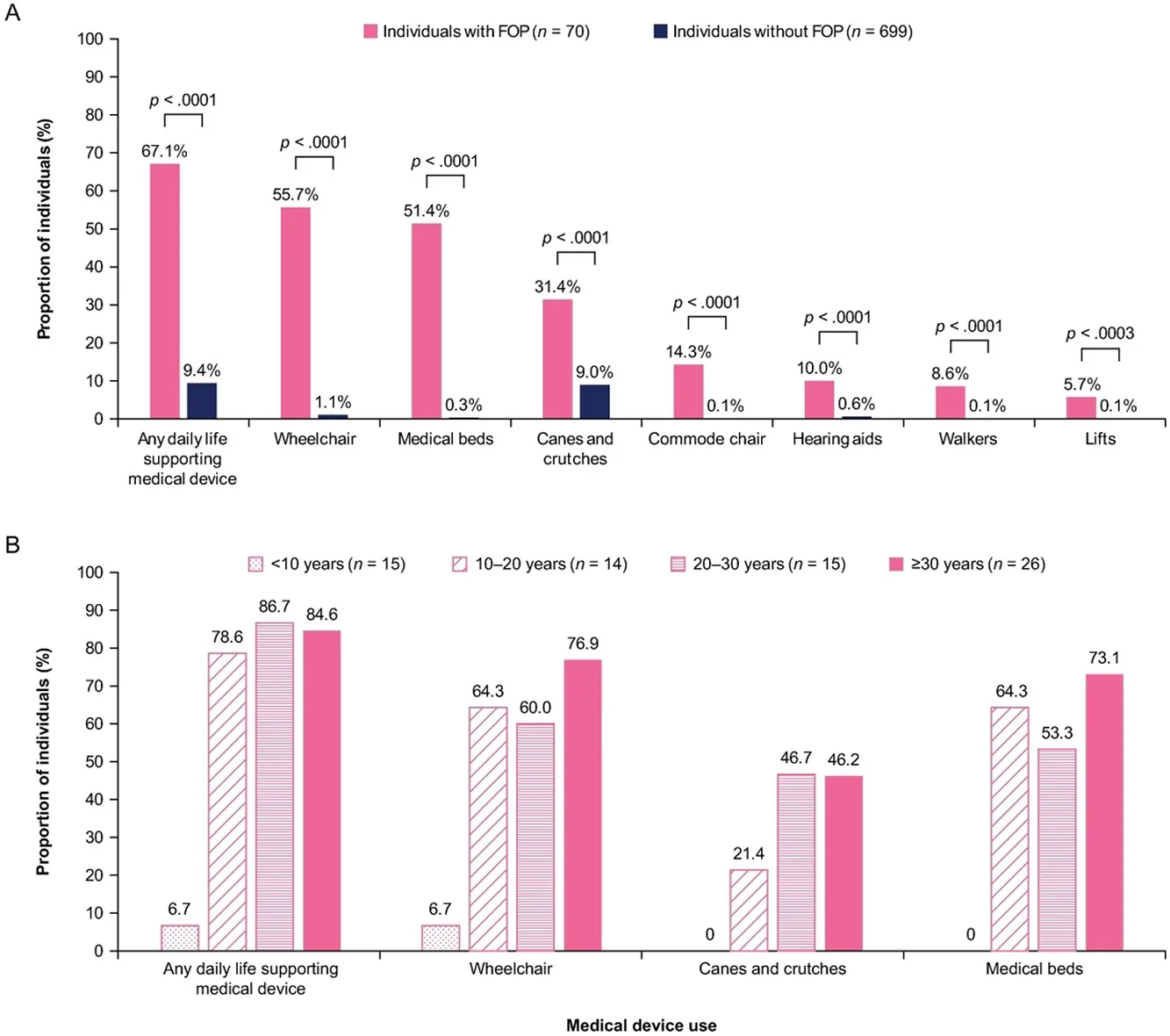
A. Medical device use in individuals with vs without FOP from 2008 to 2019. The list of medical devices of interest was defined based on the potential disabilities individuals with FOP may develop. Chi-square test, Yates’ continuity correction, or Fisher exact test was used to calculate *p*-values. Abbreviation: FOP, fibrodysplasia ossificans progressiva. B. Medical device use according to age category in individuals with FOP from 2008 to 2019. Abbreviation: FOP, fibrodysplasia ossificans progressiva.

The number of consultations with general practitioners and non-physician healthcare providers and the number of hospitalizations (Table 4 and Figure S1), as well as the use of medical devices and treatments (Figure 2A and Table S2), were significantly greater in individuals with FOP compared with controls. Regarding medical device use among individuals with FOP, the proportion of individuals using medical devices increased significantly with age for canes/crutches (*p* = .0035), wheelchairs (*p* = .0002), and medical beds (*p* < .0001; Figure 2B).

**Table 4:** Clinical encounters (office visits and outpatient consultations) by individuals with FOP (*n* = 70) and age- and sex-matched controls (*n* = 699).

	Individuals with FOP (*n* = 70)	Controls (*n* = 699) ^a^	*p*-value
**General practitioner, *n* (%)**	57 (81.4)	457 (65.4)	.0065
**Mean (SD) number of consultations**	4.6 (2.8)	3.2 (2.2)	<.0001
**Median (minimum/maximum)**	4.0 (1.0/12.0)	3.0 (1.0/12.0)	
**Pediatrician (minors only), *n* (%)**	14 (50.0)	57 (20.6)	.0004
**Mean (SD) number of consultations**	2.2 (1.5)	2.1 (1.5)	.8846
**Median (minimum/maximum)**	2.0 (1.0/5.0)	2.0 (1.0/8.0)	
**Cardiologist, *n* (%)**	5 (7.1)	7 (1.0)	.0026
**Mean (SD) number of consultations**	1.2 (0.4)	1.1 (0.4)	.9002
**Median (minimum/maximum)**	1.0 (1.0/2.0)	1.0 (1.0/2.0)	
**Neurologist, *n* (%)**	3 (4.3)	9 (1.3)	.0875
**Mean (SD) number of consultations**	1.0 (−)	1.4 (0.7)	.3302
**Median (minimum/maximum)**	1.0 (1.0/1.0)	1.0 (1.0/3.0)	
**Ophthalmologist, *n* (%)**	9 (12.9)	94 (13.4)	.8900
**Mean (SD) number of consultations**	1.2 (0.4)	1.1 (0.5)	.1666
**Median (minimum/maximum)**	1.0 (1.0/2.0)	1.0 (1.0/4.0)	
**Otolaryngologist, *n* (%)**	11 (15.7)	27 (3.9)	.0003
**Mean (SD) number of consultations**	1.4 (0.7)	1.3 (0.7)	.6028
**Median (minimum/maximum)**	1.0 (1.0/3.0)	1.0 (1.0/4.0)	
**Dentist, *n* (%)**	– (–)	12 (1.7)	.6149
**Mean (SD) number of consultations**	– (–)	1.2 (0.4)	NA
**Median (minimum/maximum)**	– (–)	1.0 (1.0/2.0)	
**Pneumologist, *n* (%)**	1 (1.4)	10 (1.4)	1.0000
**Mean (SD) number of consultations**	1.0 (−)	1.1 (0.3)	1.0000
**Median (minimum/maximum)**	1.0 (1.0/1.0)	1.0 (1.0/2.0)	
**Hospital physician, *n* (%)**	36 (51.4)	112 (16.0)	<.0001
**Mean (SD) number of consultations**	2.1 (1.3)	2.0 (1.5)	.2347
**Median (minimum/maximum)**	2.0 (1.0/7.0)	1.0 (1.0/9.0)	
**Nurse consultations, *n* (%)**	29 (41.4)	108 (15.5)	<.0001
**Mean (SD) number of consultations**	4.0 (5.7)	1.8 (1.5)	.0530
**Median (minimum/maximum)**	2.0 (1.0/26.0)	1.0 (1.0/13.0)	
**Physiotherapist consultations, *n* (%)**	42 (60.0)	65 (9.3)	<.0001
**Mean (SD) number of consultations**	9.7 (5.5)	3.7 (3.0)	<.0001
**Median (minimum/maximum)**	12.0 (1.0/26.0)	3.0 (1.0/14.0)	

Abbreviation: FOP, fibrodysplasia ossificans progressiva.

^a^Controls did not have FOP.

Based on equipment reimbursement costs, it was possible to estimate the severity of disability for individuals with FOP (no walker/wheelchair required: no severity; walker required: low severity; wheelchair required: high severity). According to these definitions, approximately half of all individuals with FOP (55.7%; *n* = 39/70) had a high severity of disability, whereas 37.1% (*n* = 26/70) had no identified severity between 2008 and 2019. The proportion of individuals with disability categorized as “no severity” decreased with age, while the proportion of individuals with disability categorized as “high severity” increased with age with most individuals aged ≥30 yr assigned to this category (*p* < .0001; Figure S2).

**Figure 3:**
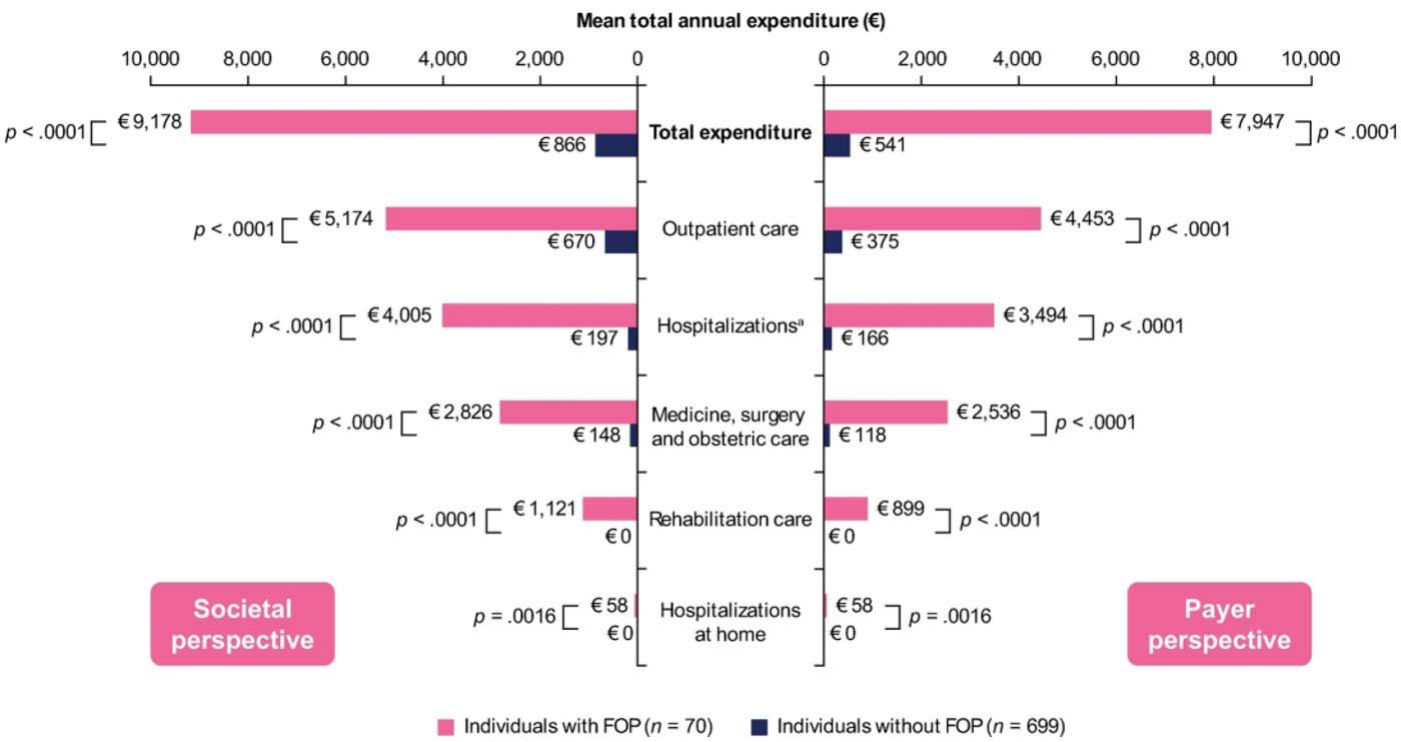
Annual healthcare expenditure from societal and payer perspectives in individuals with vs without FOP in 2019. When distribution was close to normal (non-significant Shapiro–Wilk test), a Student’s *t* test or ANOVA was used to calculate *p*-values. If this was not the case, non-parametric tests (Wilcoxon, Kruskal-Wallis tests) were used. Expenditure from a payer perspective is limited to amounts reimbursed by the French health insurance; expenditure from a societal perspective represents the total reimbursed and out-of-pocket expenses. ^a^Included hospitalizations for medical, surgical, and obstetric care; for rehabilitation; and for hospitalizations at home. Abbreviation: FOP, fibrodysplasia ossificans progressiva.

The mean total annual healthcare expenditure was more than 10 times greater in individuals with FOP than in those without FOP from a societal perspective and 14 times greater from a payer perspective (Figure 3). Total societal costs were largely driven by outpatient costs, of which medical devices were the most expensive component (mean annual cost: €1761).

All annual societal cost categories increased significantly with increasing disability, except for hospitalization costs. Mean total expenditures for individuals with no identified severity were €3152 compared with €11 820 for individuals with high disability severity level (*p* = .0002), and mean outpatient expenditures for individuals with no identified severity were €1672 compared with €7920 for those with high disability severity level (*p* < .0001). In contrast, mean cost of hospitalizations was €1480 for individuals with no identified severity and €3900 for those with high disability severity level (*p* = .3257). Outpatient costs were the only societal cost category to increase significantly with age (<10 yr, €1399; >30 yr, €7438; *p* = .0133).

### Overall survival

**Figure 4:**
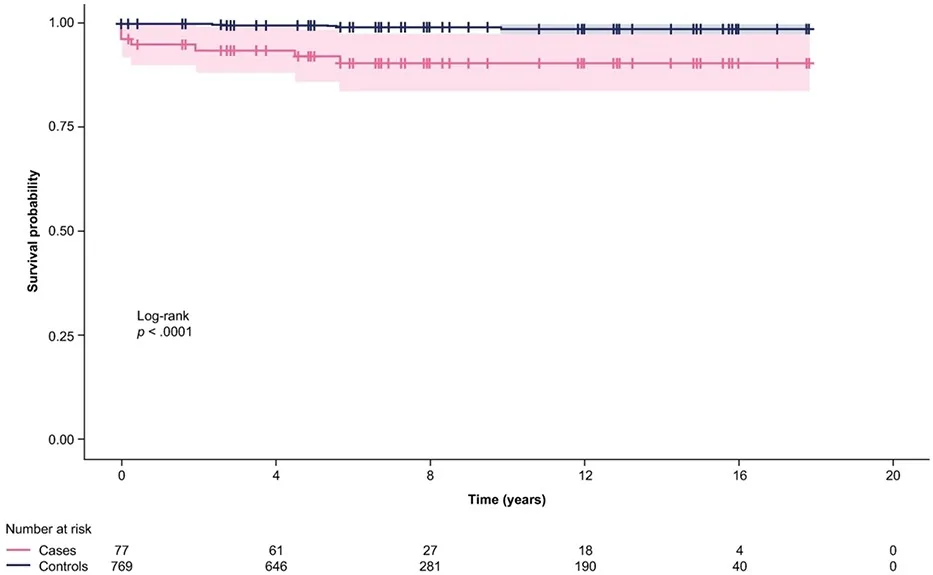
Survival of individuals with FOP compared with the control group without FOP. Data was censored on January 1, 2023. Abbreviation: FOP, fibrodysplasia ossificans progressiva.

Overall survival was significantly worse in individuals with FOP vs the control group (*p* < .0001; Figure 4). Seven deaths were reported in the 77 individuals with FOP, and 8 deaths were reported in the 769 individuals without FOP. The mean (SD) age at death for individuals with FOP and controls was 50.4 (20.9) yr and 63.1 (18.3) yr, respectively (*p* = .2239).

## Discussion

This observational, retrospective case-control comparison adds to the growing body of evidence that FOP has significant clinical, economic, and societal impacts. First, this study determined that the prevalence of FOP in France is approximately 1.4 per million inhabitants, which aligns with other estimates.^26^^,^^27^ France is unique in that it has had a network of reference centers since 2005, and since 2008 has used the BNDMR to systematically record all individuals consulting these expert sites, with a robust deduplication system to merge fragmented files and eliminate duplicate patient records from different centers for the same patient.^36^ Thus, the BNDMR has several important strengths to assist in the calculation of FOP prevalence, including its nationwide nature, its integration into hospital information systems for improved data completeness compared with standalone data sources, and its specific focus on rare disease data collection.^29^ In contrast, the question of the worldwide prevalence of FOP is challenging to answer, and it is unclear whether the prevalence rate is consistent across continents and countries. A study based on expert sites in several countries carried out by the IFOPA in 2016 showed very heterogeneous prevalence rates, ranging from 0.04 per million registered and confirmed individuals with FOP in the Asia-Pacific region to approximately 0.65 per million in North America.^37^ Additional studies of FOP prevalence are needed to gain a clearer understanding of the global impact of FOP and to explore potential geographic or environmental modifiers that may have an impact on the regional prevalence of FOP.

According to our findings, individuals with FOP have an increased risk of early mortality and additional morbidity compared with a control population of age- and sex-matched individuals with any disease other than FOP. The difference in mean age at death for individuals with FOP vs controls was more than 10 yr, although this difference was not statistically significant. As this control group included individuals from the SNDS with any disease other than FOP and with underlying health conditions, it is not representative of a healthy population. Thus, it is likely that the observed difference in life expectancy in this study is underestimated compared with the general population. For comparison, a 2010 review of patient registries from the IFOPA and the International FOP Clinic at the University of Pennsylvania revealed a median life expectancy of 56 yr for individuals with FOP vs an average life expectancy at birth of 77.5 yr in the US (calculated in 2024, based on 2022 data) and 81.4 yr in the European Union (calculated in 2025, based on 2023 data).^38^^,^^39^

In the current study, comorbidities, such as malnutrition, depression, urolithiasis, and respiratory failure, were more common in individuals with FOP than in the age- and sex-matched control population. Malignant tumors occurred in 3 (4.3%) individuals with FOP, which was significantly higher than in the control group (0.9%; *p* = .0407) as well as among French adolescent and young adult populations (0.035%-0.058%).^40^^,^^41^ Details about the malignant tumors and confirmation by the treating physician were not available for analysis in this study; notably, however, FOP can often be misdiagnosed as cancer or other conditions such as ankylosing spondylitis.^42^^,^^43^ The authors noted that the 2 individuals with comorbidities of ankylosing spondylitis were likely cases of mistaken coding entered into the BNDMR, further emphasizing the need for healthcare providers to improve their understanding and recognition of FOP. Other studies have shown that individuals with FOP are at risk of additional health issues such as fractures, severe restrictive lung disease, heart disease, scoliosis, pressure ulcers, severe weight loss due to jaw ankylosis, gastrointestinal issues, hearing impairment, thrombosis, and acute and chronic pain.^1^^,^^8^^,^^25^^,^^44^ Taken together, the available evidence suggests that individuals with FOP face a substantially reduced life expectancy and higher concomitant health risks compared with controls without FOP. In addition, individuals with FOP may encounter inappropriate, invasive, and unnecessary prediagnostic procedures (eg, biopsies or other medical interventions) as well as nonessential surgeries after diagnosis despite guidance from the International Clinical Council on FOP.^45^ These harmful medical interventions could accelerate disease progression and may lead to permanent loss of mobility because of trauma-induced ossification.^26^^,^^46^

Compared with controls in this study, individuals with FOP had greater HCRU, medical treatments, hospitalizations, and use of medical devices. In a baseline analysis of the IFOPA global patient-reported registry, approximately 86% of all participants reported they had at least 1 visit with a physician and 36% had more than 5 visits.^8^ Similarly, in an international survey of individuals with FOP and their family members assessing lived experiences, most individuals with FOP reported utilization of general practitioner and dental services over the previous 12 mo (78% and 59%, respectively), rates that were comparable to those observed in the general population (83% and 63%, respectively) as noted in another study.^25^ Individuals with FOP also reported substantial use of aids, assistive devices, care assistant services, and adaptive equipment to support mobility and self-care, as well as participation in work, education, and recreational activities.^25^ Caregiver dependence was also high, with many requiring daily support and medical oversight for disease management and flare-up prevention.^25^ Furthermore, reported medication use in this group ranged from 18% to 40% for symptoms of depression, anxiety, sleeping problems, hypertension, and pain.^25^

As FOP is a progressive disease, the cumulative and irreversible HO leads to increasing disability with increasing age.^12^ The median number of aids, assistive devices, and adaptations used by individuals with FOP in the current study was 2 for participants aged <9 yr, increasing to 13 for participants aged >15 yr. This observed decrease in mobility with increasing age in the current study confirms the findings from the patient and caregiver survey, in which mobility and joint function declined with age.^25^ Increasing joint mobility issues, as measured using a Patient-Reported Mobility Assessment (PRMA), were also associated with increased HCRU.^25^ This reinforces the burden of disease progression and highlights the need for interventions that may slow functional decline, particularly in pediatric patients.

Healthcare costs in the current study were substantially higher for individuals with FOP compared with individuals with any disease other than FOP (10-fold higher from a societal perspective and 14-fold higher from a payer perspective). Most of the cost burden was driven by outpatient care and acute-care hospitalizations. These costs reflect the higher rate of disabilities among individuals with FOP vs other diseases, even younger individuals, as indicated by greater use of medical devices such as canes/crutches and wheelchairs (approximately two-thirds of individuals had a wheelchair). Increased disability was also associated with increased societal costs. Available data on healthcare costs for individuals with FOP are scarce, but the aforementioned international survey of individuals with FOP and their family members about their lived experience reported individuals with FOP faced considerable direct and indirect costs associated with the disease.^25^ The most common cost of care areas reported were medical therapies/doctors and mobility/daily activities/pay for assistance.^25^ Mean cost of care in the 12 mo prior to survey completion was higher for those with higher PRMA total scores (reported only for individuals from the US).^25^ Thus, data for the substantial healthcare costs associated with FOP are emerging, but there is strong evidence that FOP is frequently misdiagnosed or underdiagnosed; therefore, any assessment of socioeconomic impacts of FOP likely underrepresents the true nature of the issue.^37^^,^^47^^,^^48^

In the current study, the data show trends for the first symptoms being recognized earlier in younger individuals, while the mean age of diagnosis was higher in the older age groups. Together, these results indicate that more recently diagnosed individuals had shorter delays in diagnosis, suggesting that healthcare providers’ understanding or recognition of FOP may have improved.^26^^,^^46^ In addition, the availability of easy access to molecular confirmation of FOP has contributed to earlier diagnosis. A continued focus on earlier diagnosis of FOP should remain a key factor for gaining access to prevention measures and appropriate treatment to limit disease progression and to reduce socioeconomic impacts for individuals with FOP.

### Limitations

Not all individuals with a diagnosis of FOP identified in the BNDMR were included in this analysis. While the BNDMR records almost all individuals followed up in FOP expert centers in France,^2^ complete coverage cannot be guaranteed for several reasons, including incomplete patient records and patients followed up outside FOP expert centers. However, because France has a strong referral system, almost all patients are expected to be followed in FOP expert centers. Moreover, when the study was conducted, the national health insurance number was not systematically included; consequently, not all individuals with FOP registered in the BNDMR could be linked with the SNDS.

Individuals with FOP were age- and sex-matched to individuals from the SNDS, which represents a cohort with underlying health conditions rather than a healthy population. Consequently, while we cannot estimate the socioeconomic costs of FOP compared with healthy individuals, the difference is likely greater than observed here. As such, cost differences between patients with FOP and controls should be interpreted as costs attributable to FOP, assuming other factors are equal. Finally, all comparisons should be interpreted with caution as no adjustment was made for multiple testing.

Since the SNDS only contains data about reimbursed care, self-medication, and the use of non-reimbursed over-the-counter medications and medical devices (eg, some hearing aids) and non-medical professional support (psychologists) they are not captured for individuals with FOP or the control group. Thus, the direct costs associated with these expenses are also not captured. Additionally, the study does not include frequency of consultations by age categories or by disease severity, which would be useful in determining potential differences in HCRU among these subgroups. Moreover, the survival analysis included limited data based on age and other subgroups. Further studies to determine the causes of mortality among individuals with FOP would be beneficial. Finally, the generalizability of the data is limited because of the lack of common healthcare and registration systems in other countries.

## Conclusions

This study provides updated insights and additional evidence of the considerable clinical, economic, and societal impacts of FOP following a previous national-level analysis using these French registries.^2^ The study confirmed that the prevalence of FOP in France is approximately 1.4 per million inhabitants. A comparison of patient data from the BNDMR and SNDS reveals the substantial challenges individuals with FOP face, including increased healthcare use, socioeconomic costs, and comorbidities, as well as reduced survival compared with individuals with other diseases. The research highlights that the impact on individuals with FOP, their families, and healthcare systems grows with age and disease progression. For all stakeholders in the FOP community, these findings confirm that FOP is a major driver of negative physical, emotional, and financial impacts. Closing the gaps in disease awareness and the development of new interventions to help support a reduction in these impacts should be considered key priorities for the FOP community and healthcare providers.

## Supplementary Material

Real_FOP_JManuscript_Baujat_main_text_21July2026-clean

## Data Availability

Restrictions apply to the availability of these data, since the data underlying this publication were provided by CEMKA under contract to Ipsen.

## Medical writing support

The authors thank Christina McManus, PhD, of Envision 90TEN, an Envision Medical Communications agency, a part of Envision Pharma Group, for providing medical writing support, which was sponsored by Ipsen in accordance with Good Publication Practice guidelines.
